# Technological characteristics of sodium reduced wheat bread: Effects of fermentation type and partial replacement of salt with potassium chloride

**DOI:** 10.1002/fsn3.2917

**Published:** 2022-07-07

**Authors:** Mitra Pashaei, Neda Mollakhalili‐Meybodi, Jalal Sadeghizadeh, Leila Mirmoghtadaei, Hossein Fallahzadeh, Masoumeh Arab

**Affiliations:** ^1^ Department of food science and technology School of public health Shahid Sadoughi University of Medical Sciences Yazd Iran; ^2^ Research Center for Food Hygiene and Safety Shahid Sadoughi University of Medical Sciences Yazd Iran; ^3^ Department of Food Science and Technology Faculty of Nutrition Sciences and Food Technology National Nutrition and Food Technology Research Institute Shahid Beheshti University of Sciences Tehran Iran; ^4^ Departments of biostatistics and Epidemiology School of Public Health Center for Healthcare Data Modeling Shahid Sadoughi University of Medical Sciences Yazd Iran

**Keywords:** fermentation, potassium chloride, reduced salt, wheat bread

## Abstract

Rheological, physicochemical, textural, and sensory characteristics of wheat bread prepared by potassium chloride (KCl) substitution of sodium chloride (NaCl) at different ratio (0:100, 10:90, 20:80, 30:70, 40:60, 50:50) in the presence of two different fermentation types (*Saccharomyces cerevisiae* yeast starter (YSF) and mixed fermentation based on sourdough (MFSD)) were investigated. Considering the results obtained at this study, the technological characteristics change through KCl substitution ratio which depends on the type of fermentation. In other words, the enhanced activity of microflora in MFSD‐fermented samples and decreased activity of yeast in YSF‐fermented ones have been found by increasing the ratio of KCl incorporation level. Despite the increased activity of starter microflora in MFSD‐fermented samples through increasing the KCl incorporation level, the lowest specific volume (*p <* .05) is found in SD50 (containing 50%w/w KCl in the presence of MFSD) with a quantity equal to 1.71 ± 0.47 cm^3^/g confirming its inability to restore gases. No significant difference has been found in KCl substitution levels up to 20%w/w in YSF‐fermented samples (Y20) with control (*p *≥ .05). The lowest crumb lightness (L*) (65.27 ± 0.12), highest cohesiveness (1.31 ± 0.07 mm), and springiness (0.76 ± 0.01) is also found in Y20. Considering sensory characteristics perception, no significant difference has been found in textural characteristics perception of Y10 and Y20 containing KCl at 10%w/w and 20% w/w, respectively, in the presence of YSF with control sample (Y). The overall acceptability is also found to be more influenced by texture perception(*r* = 0.827, *p* < .00).

## Graphical abstract

Regarding color, taste, texture, and overall acceptability, it seems overall acceptability is more influenced by texture perception than taste and color. Since no significant difference has been found in textural characteristics perception of F2 and F3 containing KCl at 10%w/w and 20% w/w, respectively, with control sample, these samples are optimal ones.

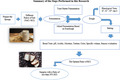

## 1 INTRODUCTION

Wheat bread is a staple food product that is considered as a rich source of starch (Onyango, 2016), proteins, vitamins, and minerals (Weegels, 2019). It is typically produced by wheat flour, water, salt, and yeast as the main ingredients. Sodium chloride (NaCl) is considered as a common salt in bread making that influences the technological, microbiological, and sensory properties such as dough development time, extensibility, yeast activity, shelf life, color, and flavor (Pasqualone et al., 2019). The common level of NaCl in bread making is about 1%–2% of total flour (Kunkulberga & Mūrniece, 2013).

Na is considered as an essential nutrient for maintaining fluid balance, cell functionality, and nerve impulses in human body (Cepanec et al., 2017). However, its high intake is related to hypertension (Bernabe‐Ortiz et al., 2020), cardiovascular disease (He & MacGregor, 2018), cancer (Banda et al., 2020), and kidney disease (Garofalo et al., 2018). Recommended daily intake of sodium intake in adults, based on the World Health Organization (WHO), is less than 2000 mg that equal to <5 g of salt (WHO, 2011). Recently, high dietary sodium intake (DSI) is considered as one of the important health concerns which are also aggravated by growing trends of using processed foods. Processed foods are the main source of sodium (about 70%–75% of total intake) in human diet (Allison & Fouladkhah, 2018), in which bread is accounted for about 30% of the overall daily intake (Avramenko et al., 2018). The detrimental impact of bread despite its low salt content is induced by its high total intake, especially in developing countries.

Various studies were conducted on the production of low salt foods. Partial substitution of sodium with potassium is a popular strategy in the production of low sodium foods such as meat, dairy, and bakery products (Antúnez et al., 2018; Arab et al., 2019; Zheng et al., 2019). Adequate daily intake of potassium has positive effects on the reduction of blood pressure (Filippini et al., 2020), decreasing the risk of cardiovascular diseases (Engberink et al., 2020), and age‐related bone loss (Kong et al., 2017). Regarding WHO recommendation, the daily intake of potassium needs to be at least 3510 mg (WHO, 2011). Despite the positive health effects of KCl, its high usage in food products may lead to a creature of off‐flavor (bitterness and metal flavor). Adding food permissible flavor enhancers, such as yeast extracts, is considered as a recommended strategy for masking the unpleasing tastes (Sinesio et al., 2019).

In addition to the ingredients, fermentation is also considered as a key step in bread baking that influences the technological and sensory characteristics of final products. Fermentation with *Saccharomyces cerevisiae* and/or mixed fermentation based on sourdough (MFSD) are commonly used techniques in bread making that produce different types of aroma and flavoring agents. To the best of our knowledge, no study is available on the characterization of wheat bread as the effects of partial replacement of NaCl with KCl and fermentation types (*S. cerevisiae* and/or MFSD). Therefore, the purpose of this work was developing the wheat bread with different ratios of NaCl/KCl and yeast and/or MFSD dough fermentation and evaluating the color, rheological, textural, and sensory characteristics of the final products.

## 2 MATERIALS AND METHODS

### 2.1 Materials

This research was a lab‐trial experimental study. Commercial wheat flour (13.95% w w^‐1^ moisture, 0.23% w w^‐1^ ash, and 10.93% w w^‐1^ protein) was purchased from Karaj Etthd company. Sodium chloride (NaCl) and potassium chloride (KCl) were prepared by Golha Company (Tehran, Iran) and Merk Company (purchased with scientific retail), respectively. The other constituents of wheat bread (active dry yeast (Khuzestan company), sugar, canola oil) were purchased from a local supermarket. All chemical reagents were also prepared by Merck Company.

### 2.2 Dough preparation

The effect of different KCl substitution levels and two fermentation types (*S. cerevisiae* yeast and MFSD) as presented in Table 1, was investigated. After determining the amount of water absorption, the formulation of two types of fermentation used in this study was prepared as follows.

**TABLE 1:** Treatment studied in the present study

Sample	Independent variables		Abbreviation
	Type of fermentation	Salt proportion (NaCl: KCl) (% w w^−1^)	
F1	Yeast (*S. cerevisiae*)	100:0	Y
F2	Yeast (*S. cerevisiae*)	90:10	Y10
F3	Yeast (*S. cerevisiae*)	80:20	Y20
F4	Yeast (*S. cerevisiae*)	70:30	Y30
F5	Yeast (*S. cerevisiae*)	60:40	Y40
F6	Sourdough	50:50	Y50
F7	Sourdough	90:10	SD10
F8	Sourdough	80:20	SD20
F9	Sourdough	70:30	SD30
F10	Sourdough	60:40	SD40
F11	Sourdough	50:50	SD50

Note

Y and SD abbreviations are representative of *S. cerevisiae* yeast starter and mixed fermentation based on sourdough fermentation types, respectively.

#### 2.2.1 Yeast starter fermentation

In order to prepare yeast starter fermentation (YSF) dough, the straight dough method was used. The dough was prepared using wheat flour, 1% w/w salt (different ratio of NaCl/KCl), 0.5%w/w sugar, 3% canola oil, and 2.2% w/w active dry yeast. The mixture was blended with appropriate amounts of potable water as determined by Farinograph test. Afterward, the mixed ingredients were fermented at 29 ± 0.5°C for 4 h. Then, the dough was divided into 300 g pieces and baked at 220°C for 30 min in a convection oven (Model PFB‐2, Duke manufacturing Company, St Louis, MO, USA) (Gamel et al., 2015).

#### 2.2.2 Mixed fermentation based on sourdough

Sponge‐dough breads were prepared with a proportion of 60:40 (sponge:dough). The sponge was prepared by 60%w/w wheat flour and 60% w/w water (as estimated by Farinograph test). Afterward, the mixture was held for 20 h at 29 ± 0.5°C. The dough was prepared by blending residual wheat flour (40%) and water with salt, sugar, canola oil, and active dry yeast (at above‐mentioned percentage) and incubated at 29 ± 0.5°C for 4 h. Bread baking was done like above‐mentioned processes (Gamel et al., 2015).

### 2.3 Physicochemical characteristics

#### 2.3.1 Dough pH and titratable acidity

pH meter (thermoOrion Model 420A) was used for the evaluation of dough pH. Titratable acidity was estimated by titration with NaOH 5 Mm and calculated as mg lactic acid to g of fresh dough. For the measurements, 5 g of dough was dissolved in 10 ml of deionized water at room temperature (21 ± 1°C) (Gamel et al., 2015).

#### 2.3.2 Specific volume

The canola seed replacement method was used for determining the loaf volume of breads. The specific volume was accounted by dividing the bread volume to the weight, approximately 1 h after leaving the oven (Moradi et al., 2020).

#### 2.3.3 Moisture content

The oven drying method was applied to the determination of moisture content of the bread samples. The samples were dried in an air oven (at 105 ± 0.05°C) until the differences in two weighing (at 15 min intervals) were less than 0.1% w/w. The following equation was used for moisture determination (Lu et al., 2014):

$$\text{Moisture content}\;=\frac{\text{M sample}‐\text{M after drying}}{\text{M sample}}\times 100\text{\textbackslash{}\%}\;$$

### 2.4 Color determination

The color of bread was determined by Hunter Lab instruments (D25‐9000 made in Germany). L* (lightness), a*(redness/greenness), and b* (yellowness/blueness) values were measured for evaluating the color of bread one day after baking (Esmaeilifard et al., 2017):

### 2.5 Textural analysis of breads

The texture profile analyzer (TA20., KOOPA, Iran) was used for the determination of textural properties (hardness, cohesiveness, springiness, and chewiness) of bread. A 43‐mm cylinder probe using a 5‐kg loading cell (at speed of 1 mm s^−1^) was used to press a piece of the crumb (20 × 20 × 25 mm) to 50% of its original height. The tests were done at room temperature (25 ± 3°C) and six replicates (Katina et al., 2006).

### 2.6 Sensory evaluation

Nine‐point hedonic scale (1: very unpleasant and 9: extremely pleasant) was performed for sensory evaluation of bread. Thirty semi‐trained panelists (50% men and 50% women, aged from 18 to 40) were participated in sensory evaluation for ranking flavor, color, texture, and overall acceptability of the samples. Water was served between each evaluation (Menon et al., 2015).

### 2.7 Rheological measurement

A controlled shear/stress rheometer (Anton Paar MCR301, GmbH, Germany) with parallel plate geometry was used for rheological measurement. The linear viscoelastic region was estimated by strain sweep test at a strain range from 0.001% to 100% and a constant frequency of 15 Hz. The frequency sweep test was performed at a range of 0.1–100 Hz and a constant strain of 0.01%. All tests were done at 30°C. The following equations were used for determining the damping factor (tan δ) and complex modulus (G*) (Upadhyay et al., 2012):

$$\tan\delta =G^{\prime \prime }/G^{\prime }$$

$$G^{*}=\sqrt{G^{\prime 2}+G^{\prime \prime 2}}$$

### 2.8 Statistical analysis

Samples were prepared in triplicate. Descriptive statistics using mean and standard deviation were used to describe quantitative characteristics. Data analysis was done using SPSS statistical software (SPSS Statistics 23.0, Chicago, IL, USA) with two‐way ANOVA which the KCl incorporation level and fermentation type were estimated as independent variables. Homogeneous groups were determined by Tukey's post `hoc test and nonparametric test (Kruskal–Wallis test) was used for sensory evaluation. Statistical analysis in this study was based on a significance level of 95% (*p <* .05).

## 3 RESULTS AND DISCUSSION

### 3.1 Physicochemical characteristics of wheat bread

The physicochemical characteristics of wheat bread influenced by substitution ratio of potassium chloride (KCl) and fermentation type are presented in Table 2. The pH value is represented to be in the range of 5.35–6.37. The lowest and highest pH has been observed in SD50 and Y50 samples, respectively. However, significantly lower pH and increased acidity have been found in MFSD‐fermented samples (*p <*.05), its change trend according to KCl substitution ratio is dependent on the fermentation type. In other words, while a decrease has been found in pH value of MFSD‐fermented samples, it has been increased in yeast starter‐fermented ones. Increasing the KCl substitution ratio has been found to increase the activity of lactic acid bacteria, and inhibit the activity of yeasts (Gan et al., 2021).

**TABLE 2:** Physicochemical properties of wheat bread prepared in the present study

Sample	Parameters			
	pH (−)	Acidity (TTA)	Specific volume (cm^3^ g^−1^)	Moisture (%)
Y	5.73 ± 0.01^d^	0.15 ± 0.07^f^	3.21 ± 0.12^a^	39.96 ± 0.08^a^
Y10	5.79 ± 0.02^c^	0.2 ± 0.05^e^	3.02 ± 0.53^a^	38.64 ± 1.01^a^
Y20	5.69 ± 0.01^d^	0.2 ± 0.15^e^	3.36 ± 0.09^a^	39.96 ± 0.07^a^
Y30	6.32 ± 0.01^b^	0.3 ± 0.09^d^	2.65 ± 0.26^b^	39.52 ± 0.09^a^
Y40	6.36 ± 0.02^a^	0.2 ± 0.11^e^	2.98 ± 0.63^ab^	41.21 ± 1.09^a^
Y50	6.37 ± 0.02^a^	0.2 ± 0.07^e^	2.84 ± 0.54^ab^	39.04 ± 1.04^a^
SD10	5.61 ± 0.01^e^	0.5 ± 0.12^b^	2.65 ± 0.22^ab^	33.46 ± 0.08^a^
SD20	5.70 ± 0.02^d^	0.2 ± 0.06^e^	2.42 ± 0.24^b^	36.14 ± 0.06^a^
SD30	5.51 ± 0.01^f^	0.6 ± 0.05^a^	2.29 ± 0.07^bc^	32.51 ± 1.06^a^
SD40	5.48 ± 0.01^f^	0.4 ± 0.13^c^	2.24 ± 0.12^bc^	29.60 ± 1.04^b^
SD50	5.35 ± 0.01^g^	0.5 ± 0.8^b^	1.71 ± 0.47^c^	31.77 ± 1.05^b^

Note

Data are reported as average ± standard deviation. Values with different lowercase letters according to Tukey's test are significantly different in each column (*p <* .05).

KCl incorporation level at 20%w/w has been reported to be necessary for the correct activity of yeast (Yenush, 2016). Increasing the KCl incorporation ratio in MFSD‐fermented samples, however increased the carbon dioxide production ratio by facilitating the activity of lactic acid bacteria, and decreased its specific volume by decreasing the gas retention capacity. The lowest specific volume is found in SD50 (*p <*.05) which is equal to 1.71 ± 0.47 cm^3^ g^−1^. The specific volume is considered as an indicator of dough's potential to retain and expand gases produced through fermentation (Kaur et al., 2011). The controversial finding has been observed about the impact of MFSD on the specific volume of wheat bread which is dependent on the gluten degradation degree (Loponen et al., 2004). It is hypothesized that the gluten degradation degree has been increased by increasing the KCl incorporation level in MFSD‐fermented samples.

Respecting moisture content which was not significantly influenced by fermentation type and KCl incorporation level, it has been significantly decreased in SD40 and SD50 samples (*p <* .05). Regarding Hofmeister series, the hydration capacity of K^+^ has been decreased compared to Na^+^ resulting in its higher diameter and lower charge to diameter ratio (Simsek & Martinez, 2016). Despite an assumed decrease in moisture content of yeast‐fermented samples in the presence of high incorporation ratio of KCl, it's no significant change may be attributed to its lower yeast activity (Spina et al., 2015), lower specific volume, and decreased migration of water through baking (Roman et al., 2020). A decreased water migration by decreasing specific volume has also been found by Duduet al. (2020), attributed to reduced water availability in the dough, which reduces the binding of starch and gluten and does not form a strong network of gluten with high gas retention capability (Dudu et al., 2020; McCann & Day, 2013).

### 3.2 Color analysis

The crumb and crust color analysis of wheat bread has been determined using CIE L*a*b* scale as demonstrated in Table 3. Results indicated that the substitution ratio of KCl and fermentation type can significantly influence the color parameters of wheat bread. Regarding the crumb color, the highest and lowest lightness has been found in SD20 and control samples with L* values equal to 70.73 ± 0.10 and 64.88 ± 0.16, respectively. However, in KCl replaced samples, the lowest L* is found in Y10 and Y20 with no significant difference (*p ≥* .05). The results about the highest and lowest redness of crumb color have been related to SD50 and Y20 samples with a* value equal to 1.64 ± 0.06 and 0.51 ± 0.06, respectively. The highest and lowest b* has been found in SD50 and SD20 with values equal to 21.31 ± 0.09 and 18.44 ± 0.07, respectively. As the color characteristics of crumb are generally determined by its formulation and structure‐forming characteristics (Conforti & Davis, 2006), it seems that the higher gas production capacity of yeast in the presence of 20% w/w KCl (Yenush, 2016), will increase the size and amount of air bubble and light scattering and consequently decrease its L* value (Beikzadeh et al., 2018). Considering the SD20 sample, the lowest b* is also found simultaneously. In other words, the highest lightness is accompanied by the lowest blueness which is comparable to the findings of Shittu et al. (2009).

**TABLE 3:** Color analysis of crust and crumb of prepared wheat bread samples in the present study

Sample	Crust color			Crumb color		
	L*	a*	b*	L*	a*	b*
Y	50.25 ± 0.05^e^	14.85 ± 0.01^d^	34.11 ± 0.24^c^	64.88 ± 0.16^e^	0.68 ± 0.03^g^	18.61 ± 0.06^e^
Y10	53.78 ± 0.07^c^	12.99 ± 0.05^f^	32.01 ± 0.05^d^	65.87 ± 0.05^e^	0.75 ± 0.01^de^	19.36 ± 0.11^d^
Y20	72.84 ± 0.67^a^	5.40 ± 0.17^g^	22.18 ± 0.06^f^	65.72 ± 0.12^e^	0.51 ± 0.06^f^	19.23 ± 0.02^d^
Y30	53.80 ± 0.56^c^	16.39 ± 0.14^b^	31.99 ± 0.24^d^	68.63 ± 0.17^b^	0.90 ± 0.08^d^	21.17 ± 0.04^a^
Y40	50.23 ± 0.09^e^	17.33 ± 0.02^a^	32.49 ± 0.33^d^	69.27 ± 0.05^a^	0.70 ± 0.06^e^	20.45 ± 0.06^b^
Y50	52.87 ± 0.70^d^	14.31 ± 0.01^e^	36.25 ± 0.36^b^	68.85 ± 0.07^b^	0.87 ± 0.04^de^	20.80 ± 0.15^b^
SD10	50.04 ± 0.08^e^	15.52 ± 0.06^c^	32.62 ± 0.02^d^	66.28 ± 0.07^d^	1.47 ± 0.01^ab^	19.18 ± 0.04^d^
SD20	54.75 ± 0.45^b^	14.63 ± 0.14^de^	31.04 ± 0.09^e^	70.73 ± 0.10^a^	0.92 ± 0.07^d^	18.44 ± 0.07^e^
SD30	55.96 ± 0.60^b^	14.81 ± 0.10^d^	33.97 ± 0.35^c^	68.15 ± 0.09^b^	1.17 ± 0.08^c^	20.22 ± 0.10^c^
SD40	53.52 ± 0.71^c^	16.34 ± 0.17^b^	34.51 ± 0.11^c^	67.19 ± 0.04^c^	1.45 ± 0.09^b^	20.68 ± 0.04^b^
SD50	52.53 ± 0.76^d^	14.14 ± 0.15^e^	38.91 ± 0.37^a^	65.60 ± 0.17^e^	1.64 ± 0.06^a^	21.31 ± 0.09^a^

Note

Data are reported as average ± standard deviation. Values with different lowercase letters according to Tukey's test are significantly different in each column (*p <*.05).

Regarding the crust color analysis, the highest and lowest lightness is found in Y20 and SD10 samples with L* values equal to 72.84 ± 0.67 and 50.04 ± 0.08, respectively. The results about the highest and lowest redness of crust color have been related to Y40 and Y20 samples with a* value equal to 17.33 ± 0.02 and 5.40 ± 0.17, respectively. The highest and lowest blueness has been found in SD50 and Y20 with b* value equal to 38.91 ± 0.37 and 22.18 ± 0.06, respectively.

The lowest a* value is also found in Y20 containing 20% w/w KCl in the presence of YSF. The decrease in a* with an increase in L* in crust color is also found by (Shittu et al., 2008), which is attributed to the Maillard reaction. The inability of KCl incorporation at 20%w/w in the prevention of yeast activity and subsequent reduction of substrate access (Thiele et al., 2002), has reduced the Maillard reaction (Spina et al., 2015). Considering the impact of fermentation type on crust color analysis of wheat bread, no significant difference has been found (*p *≥ .05). However, the crust color is deeply dependent on the Maillard reaction products (Pashaei et al., 2021) and despite the facilitating mechanism of MFSD in providing the substrate of the Maillard reaction (Thiele et al., 2002), it seems that ascending activity of lactic acid bacteria in the presence of KCl may even lead to a decrease in substrate of Maillard reaction with no significant impact in appearance perception of the final product (*p* ≥ .05). Increasing the amount of exopolysaccharides as a result of intensifying the activity of MFSD further reduces sugars and consequently brightens the bread crust and crumb (Di Monaco et al., 2015). However, this assumption needs further investigation.

### 3.3 Texture profile analysis

The textural characteristics of wheat breads prepared by incorporation of KCl at different ratios and different fermentation types are presented in Table 4. The lowest and highest hardness is observed in Y and SD50 with values equal to 225.57 ± 0.07 and 1026.80 ± 0.09 g, respectively. The hardness parameter has been influenced by fermentation type and substitution ratio of KCl significantly (*p <* .05). Using MFSD has been shown to enhance the hardness depending on the ratio of KCl incorporation level. In other words, increasing the KCl ratio make the hardness more prone to fermentation type in a way that while 64.84% increase has been observed by MFSD at formulations containing 10% w/w KCl, it has been increased by 175.84 at 50% w/w KCl containing ones. The enhanced activity of lactic acid bacteria (Gan et al., 2021) and decreased activity of yeast (Spina et al., 2015) in samples containing a high quantity of KCl is considered as the main reason which directly influences the gas retention capacity and gas production capability and consequently the specific volume and hardness of wheat bread (Gan et al., 2021; Linko et al., 1984). In wheat‐based products, the textural characteristics are strictly dependent on the formation of the gluten network with the ability to be extended and restore gases to contribute to the formation of a cellular crumb structure (Katina et al., 2005).

**TABLE 4:** Texture analysis of prepared wheat bread samples in the present study

Sample	Parameters			
	Hardness (g)	Springiness (mm)	Cohesiveness (−)	Chewiness (mJ)
Y	225.57 ± 0.07^h^	1.30 ± 0.06^a^	0.76 ± 0.04^a^	172.49 ± 0.04^i^
Y10	288.87 ± 0.06^g^	1.15 ± 0.07^b^	0.74 ± 0.04^ab^	246.27 ± 0.04^h^
Y20	382.74 ± 0.06^e^	1.31 ± 0.07^a^	0.76 ± 0.01^a^	399.96 ± 0.05^c^
Y30	474.28 ± 0.08^d^	1.05 ± 0.01^c^	0.67 ± 0.04^ab^	321.13 ± 0.05^ef^
Y40	385.95 ± 0.09^e^	1.23 ± 0.06^ab^	0.69 ± 0.03^ab^	309.15 ± 0.04^f^
Y50	372.24 ± 0.08^f^	1.11 ± 0.05^b^	0.67 ± 0.03^ab^	271.76 ± 0.04^g^
SD10	476.20 ± 0.08^d^	0.85 ± 0.06^d^	0.67 ± 0.05^ab^	271.61 ± 0.01^g^
SD20	711.01 ± 0.06^b^	0.85 ± 0.04^d^	0.67 ± 0.02^ab^	404.19 ± 0.06^b^
SD30	707.18 ± 0.06^b^	0.78 ± 0.01^e^	0.59 ± 0.02^c^	329.30 ± 0.06^e^
SD40	650.84 ± 0.07^c^	0.81 ± 0.05^de^	0.64 ± 0.03^b^	337.13 ± 0.05^d^
SD50	1026.80 ± 0.09^a^	0.76 ± 0.02^e^	0.6 ± 0.01^bc^	445.32 ± 0.05^a^

Note

Data are reported as average ± standard deviation. Values with different lowercase letters according to Tukey's test are significantly different in each column (*p <* .05).

Decreasing the pH value has been reported to decrease the specific volume and increase the hardness by weakening the gluten network and decreasing the gas retention capacity (Mohammadi et al., 2015; Schmiele et al., 2017). The pH differences in formulations fermented differently are more obvious at a higher level of KCl incorporation. In MFSD‐fermented samples, the increase observed in hardness compared to yeast‐fermented samples is accompanied by chewiness enhancement and a decrease in springiness and cohesiveness which is in accordance with (Karaman et al., 2018). Regarding, the weakening of the gluten network through pH decrease and the degradation induced by lactic acid bacteria are considered as the main reasons (Angioloni et al., 2006; Katina et al., 2005). In KCl‐containing samples, the highest springiness and cohesiveness (as indicators of internal band strength) are found in Y20 (with no significant difference with control (*p *≥ .05)) equal to 1.31 ± 0.07 mm and 0.76 ± 0.01, respectively.

Regarding, the highest stability of gluten network corresponding to covalent and non‐covalent crosslink formation in treated samples is found in Y20 containing KCl: NaCl ratio at 20:80 and in the presence of yeast starter. The decrease observed in cohesiveness and springiness by increasing the KCl incorporation level and changing the fermentation type may be attributed to decreased activity of yeast and enhanced activity of lactic acid bacteria. Inadequate activity of yeast starter in yeast‐fermented samples and increased hydrolysis of the gluten by lactic acid bacteria and/ or pH‐dependent activation of proteolytic enzymes in cereals in MFSD‐fermented samples are considered as the main reasons (Kumala & Sutrisno, 2020; Loponen et al., 2004; Matos & Rosell, 2012; Mollakhalili Meybodi et al., 2015).

### 3.4 Sensory analysis

**FIGURE 1:**
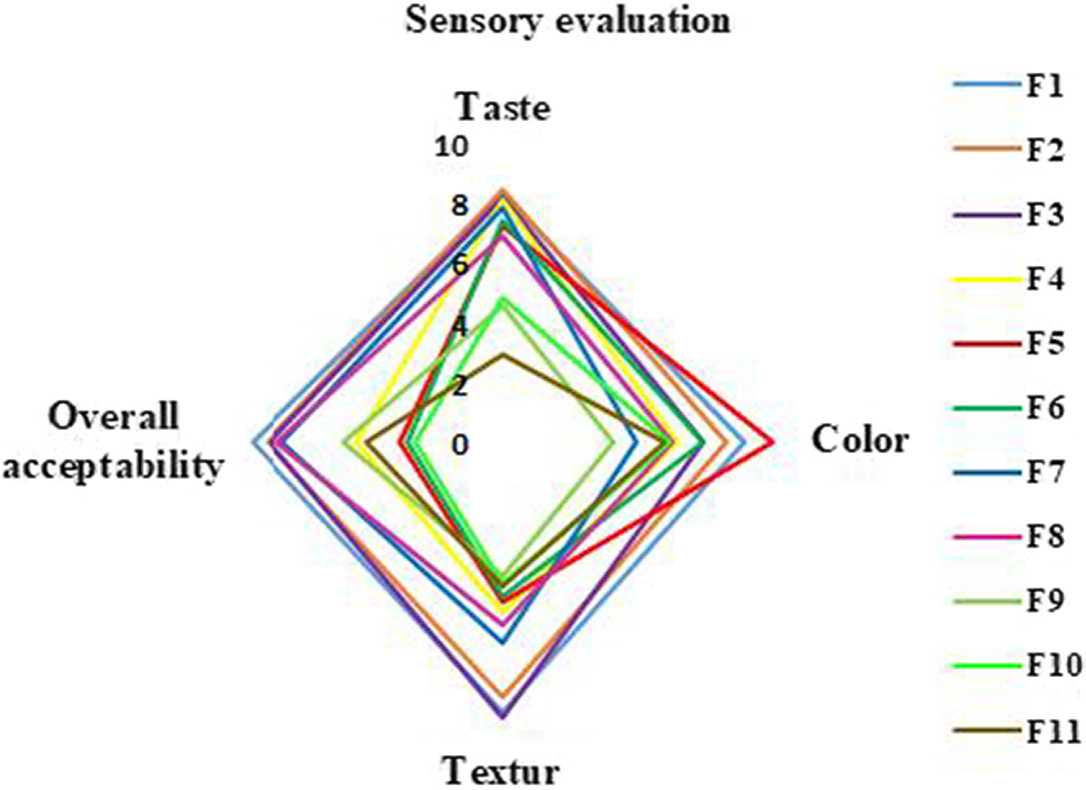
Radar plot for sensory evaluation of wheat breads containing KCl: NaCl (at different ratios) and fermentation types. Data are reported as average ± standard deviation

Regarding the importance of overall acceptability in the formulation of new products, sensory evaluation of wheat breads prepared by KCl incorporation and different fermentation methods are reported in Figure 1. Considering characteristics perceived by the consumer, the lowest acceptability in taste, color, texture, and overall acceptability is found in samples fermented by MFSD especially those containing KCl at its highest level. No significant difference has been found in textural characteristics perception of Y10 and Y20 containing KCl at 10%w/w and 20% w/w, respectively, with control sample (Y). The above‐mentioned samples have also the highest specific volume. Regarding, the raise of the specific volume seems to meet the expectation of consumers which is in accordance with (Mohammadi et al., 2021).

The color perception by a consumer which was all higher than moderate has not significantly influenced by fermentation type and KCl incorporation level (*p *≥ .05). However, it was previously stated that using MFSD significantly affects the color perception (Chiavaro et al., 2008; Coda et al., 2011; Jitrakbumrung & Therdthai, 2014; Rinaldi et al., 2015; Murtaza & Ahmad, 2007); it was not observed at this study which is in accordance with (Crowley et al., 2002). Regarding the instrumental evaluation of color, the complete consumption of the Maillard reaction substrate by lactic acid bacteria which is enhanced by increasing the KCl incorporation level is considered as the reason. This finding is in accordance with (Di Monaco et al., 2015).

The taste perception in yeast‐fermented samples was not adversely influenced by KCl incorporation level while reverse order has been found in MFSD‐fermented samples (Bolger et al., 2017). In other words, the enhanced activity of lactic acid bacteria in the presence of KCl at high content adversely changed the taste perception of bread. This finding is also observed by D Kunkulberga (Antúnez et al., 2018). Reducing the salinity taste perception has also been reported to decrease the taste preference in MFSD‐fermented samples containing a high level of KCl (Antúnez et al., 2018). Destruction of the structure by MFSD‐fermented samples, (Mohammadi et al., 2021), the difference in diameter of sodium and potassium (Simsek & Martinez, 2016), and consequently its influensive impact on reducing the water absorption content (Kaur et al., 2011) are also potentially associated with a decrease in taste. Chewiness enhancement is also reported as a reason for the decrease in taste perception of MFSD‐fermented samples (Penget al., 2017).

Regarding color, taste, texture, and overall acceptability, it seems overall acceptability is more influenced by texture perception (*r* = 0.827, *p <* .00) than taste and color which is in accordance with (Kim et al., 2017).

### 3.5 Rheological characteristics

**FIGURE 2:**
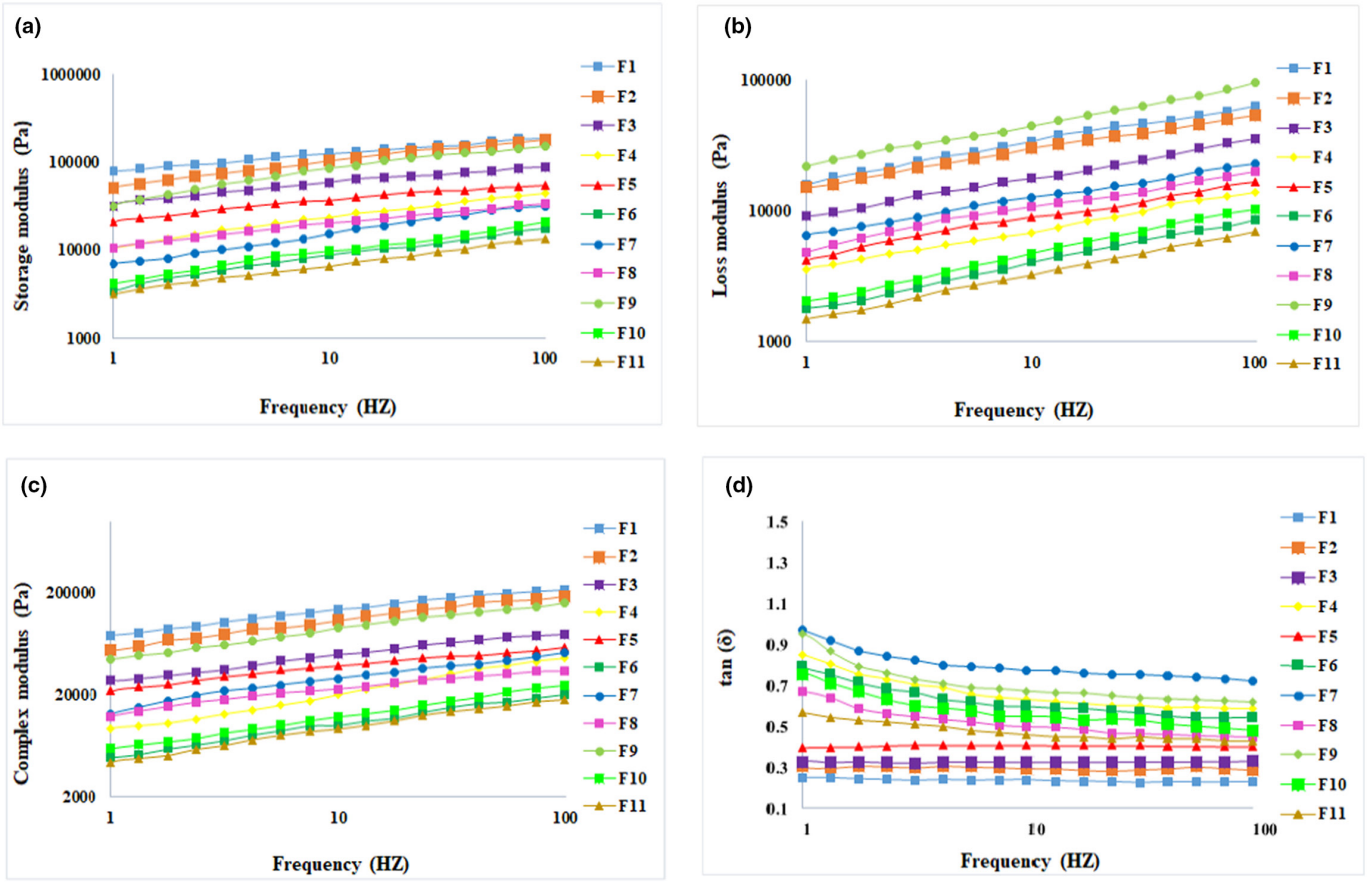
Frequency sweep curves of experimental wheat dough samples. Curves are the mean of at least two replicates. a, b, c, and d are storage modulus (*G*'), loss modulus (*G*"), complex modulus (G*), and damping factor (tan δ), respectively

Dynamic viscoelastic characteristics of wheat bread were determined by a frequency sweep test at a frequency range of 0.01–10 Hz. The frequency sweep curves of wheat dough containing different KCl: NaCl ratios and fermented differently are presented in Figure 2a–d as storage modulus (*G*'), loss modulus (*G*"), complex modulus (G*), and tan δ, respectively. As depicted in Figure 2. *G*', *G*", and G* values have increased by increasing the frequency range. All formulations were frequency dependent with storage modulus greater than loss modulus (*G*' > *G*") at a whole range of angular frequencies as an indicator of elastic‐like gel formation of wheat bread as demonstrated by Meybodi et al. (2019).

Elastic and viscose moduli are generally monitored as quality‐determining factors as high‐quality bread should be more elastic than viscose (Meybodi et al., 2019). Alongside, the complex modulus and damping factor also provide valuable information about dough strength. Considering both elastic and viscose moduli, complex modulus should be optimized as formulations with high complex modulus are generally too rigid to facilitate the growth of air bubbles and those having low G* are unable to restore gases (Mollakhalili Meybodi & Mohammadifar, 2014).

Generally, lower complex modulus and higher tan δ are found in MFSD‐fermented samples. The stronger proteolysis induced by lactic acid bacteria and its stronger acid formation resulted in gluten network degradation and consequently its lower dough strength (Belz et al., 2019; Shiri et al., 2021). Considering the impact of KCl incorporation level, a different manner has been found in samples fermented by yeast starter or mixed fermentation based on sourdough.

In yeast‐fermented samples, increasing the KCl incorporation level decreased *G*', *G*", and G* and increased the tan δ which is in accordance with (Miller & Hoseney, 1999). In other words, despite the decrease observed in both elastic and viscose moduli, the G’ modulus decreased more sharply in yeast‐fermented samples via increasing the KCl incorporation level. A decrease in dough development has been reported to result in loss modulus enhancement which is attributed to gliadin: glutenin ratio enhancement (Barak et al., 2013; Chen et al., 2018; Uthayakumaran et al., 2000; Vereijken et al., 2000). Regarding, the dominance of tan δ in samples containing higher KCl incorporation level is attributed to decreased activity of yeast in the presence of KCl which prevent the dough development (Falade et al., 2014). The decrease in specific volume in these mentioned samples verifies the lower dough development too (Barak et al., 2013). The poorer dough development by decreasing the NaCl content is also found by (Belz et al., 2019; Jekle et al., 2019; Meybodi et al., 2019).

In MFSD‐fermented samples, increasing the ratio of KCl incorporation level enhances the degradation activity of lactic acid bacteria in a way that the lowest *G*', *G*", and G* is found in SD50 contains 50% w/w KCl and fermented in the presence of MFSD. Furthermore, highest *G*', *G*", and G* is found in SD30 containing KCl: NaCl at 30:70 ratios. The lowest frequency dependency of G* is also found in SD20 confirming the formation of a strong gel structure (Demirkesen et al., 2010). In other words, since intermolecular crosslinking increases the elasticity and decreases tan δ (Mirsaeedghazi et al., 2008), increments of tan δ in yeast‐ and MFSD‐fermented samples in the presence of high percentages of KCl indicate structural degradation. MFSD can also increase tan δ by decreasing its elastic modulus (Angioloni et al., 2006; Clarke et al.,^2002^).

## 4 CONCLUSION

Partial substitution of sodium chloride with sodium‐free mineral salt (potassium chloride) in formulation of wheat bread is considered effective to conquer the problem of sodium over‐intake. However, the fermentation type has been found to play a vital role. Regarding parameters investigated in this study, it has been found that the technological characteristics change via KCl incorporation ratio is dependent on the type of fermentation. In other words, enhanced activity of microflora in MFSD and decreased activity of yeast in yeast starter‐fermented samples have been found via increasing the KCl incorporation level. Regarding the textural characteristics, it has also been concluded that the trend of changes in hardness depending on fermentation type is significantly dependent on the KCl incorporation level. In other words, while 64.48% increase in hardness has been found by MFSD fermentation process at 10%w/w containing samples, it has been increased by 175.84% in 50% w/w containing ones. From consumer's perspective, the KCl substitution level at 20%w/w in the presence of YSF process is totally comparable to the control sample.

## CONFLICT OF INTEREST

The authors declare that they have no conflict of interest.

## ETHICAL APPROVAL

This study was approved by the Institutional Review Board of School of public health, Shahid Sadoughi University of Medical Sciences. Approval ID:IR.SSU.SPH.REC.1399.253.

## CONSENT FOR PUBLICATION

All authors agree to publish.

## Data Availability

The research data are not shared.
